# The relationship between kynurenine metabolites and executive functions in patients with generalized anxiety disorder

**DOI:** 10.1192/j.eurpsy.2025.272

**Published:** 2025-08-26

**Authors:** D. F. Göy, F. Kulacaoglu Ozturk, A. Unlu, H. Eroglu Icli, M. A. Bik

**Affiliations:** 1 Department of Psychiatry, Health Sciences University Prof. Dr. Mazhar Osman Bakirkoy Research and Training Hospital for Psychiatry and Neurological Diseases, Istanbul; 2 Department of Biochemistry, Selcuk University, Konya; 3 Department of Biochemistry, Health Sciences University Prof. Dr. Mazhar Osman Bakirkoy Research and Training Hospital for Psychiatry and Neurological Diseases, Istanbul, Türkiye

## Abstract

**Introduction:**

Generalized anxiety disorder (GAD) is a common psychiatric condition characterized by excessive worry, concentration issues, and insomnia. Despite numerous studies, its neurometabolic mechanisms remain unclear.

**Objectives:**

This study aims to compare the levels of kynurenine pathway metabolites between GAD patients and healthy control groups, and to investigate the relationship between kynurenine metabolism products, executive functions, and disease severity in GAD patients.

**Methods:**

The study included 41 GAD patients and 41 healthy controls. Participants were enrolled after ruling out major depressive disorder using the Beck Depression Inventory. They then completed the Sociodemographic and Clinical Data Form, the Wisconsin Card Sorting Test (WCST), the Trail Making Test (TMT) A and B, the Digit Span Test (DST), the Verbal Fluency Test (VFT), the Stroop Test, and the State-Trait Anxiety Inventory. Venous blood samples were collected for serum metabolite measurements. Levels of kynurenic acid (KYNA), quinolinic acid (QUIN), tryptophan (TRP), 3-hydroxykynurenine (3-HK), kynurenine (KYN), and 3-hydroxyanthranilic acid (3-HAA) were measured using liquid chromatography-mass spectrometry (LC-MS).

**Results:**

We found that GAD patients performed significantly worse in terms of the number of categories completed on the WCST (p=0.014), TMT-A (p<0.001), TMT-B (p=0.015) and Stroop Test sub-scores (p<0.001) compared to the healthy control group. GAD patients had significantly higher QUIN levels and a lower KYNA/QUIN ratio (p<0.001) than the healthy control group, while the control group had a higher 3-HK/KYN ratio (p=0.008). A negative correlation was found between DST scores and 3-HAA (r = -0.311, p = 0.048), as well as between the KYNA/KYN ratio and the stroop test subscore (r = 0.368, p = 0.019). In the GAD group, we found a positive correlation between kynurenine levels and state anxiety scores (r=0.34; p=0.032). In regression analysis, the KYNA/QUIN ratio significantly reduced GAD risk (p=0.001; OR: 0.531), independent of test performance.

**Conclusions:**

Our study suggests that neurotoxic metabolites in the kynurenine-tryptophan metabolism may explain the executive function impairments observed in GAD. A key finding is that higher KYNA/QUIN ratios significantly reduce GAD risk, which is etiologically important and provides valuable guidance for future research.

**Disclosure of Interest:**

None Declared

